# The Phonograph in Hospital

**Published:** 1888-10-13

**Authors:** 


### The Phonograph in Hospital.

On October 1st a most successful conversazione was held at St. Mary's Hospital, and the great attraction of the evening proved to be Edison's phonograph. From eight o'clock to nearly two, Colonel Gourand and his assistants were busy in showing this marvellous instrument to a constant stream of visitors, and even Godfrey's band and the Glee Union failed to attract the attention given to a barrel organ heard through the phonograph. Again on Tuesday, this charitable instrument talked and sang for the benefit of St. Mary's, the charge to each of the audience being ten shillings, and the proceeds were devoted to the fund for the extension of the hospital. Unluckily Tuesday was a very wet day, so that the financial results were not so large as had been expected; still as the phonograph could only be shown properly to five people at once, those in charge of the instrument were kept fully occupied. Since the phonograph was first heard in England some six or seven years ago, it has been greatly improved by Mr. Edison, and no longer speaks in a muffled and ghostly voice. Indeed, it sang, and whistled, and declaimed with a distinctness that was startling. One record consisted of "Mary had a little lamb," repeated by first one and then another of Mr. Edison's assistants in the laboratory, and finally by the whole conclave together. The difference in each voice was very perceptible; anyone who knew the speakers could easily have recognised the voices, but to English ears the strong American accent common to them all was the strangest thing. Among the records were a piano solo, a trio of cornet, piano, and violin, and a tenor solo remarkably well sung. There were a few words from Mr. Edison delivered in a loud voice, which unconsciously called up visions of a big burly man; and there were coughs and laughter and cheers interrupting the different records which sounded weirdly human. The outlines of the construction of the instrument were clearly explained by those in charge. In speaking into it one speaks against a drumhead which vibrates to every tone, and on the drumhead is fixed a fine needle which also vibrates, and presses against a roller covered by a species of wax, on which the needle leaves an indentation according to the vibration. The cylinder on which the wax roll is placed is made to revolve by electricity. Then, the needle having left its mark all round and round the wax, it but remains to leave it lying in the indentations and revolve the cylinder at the same rate, and it gives out that it lately took in; and, what is more, will give it out whensoever and wheresoever that roll of wax is put on a similar instrument. When one of Mr. Edison's assistants here wishes to have a chat with his chum in America, he sits down before the phonograph and talks into it for a few minutes. Then he takes off the roll, and, putting it in a light wooden box made on purpose, posts it to his friend. When it reaches America the chum puts it on a phonograph there, and, sitting down before it, has the pleasure of hearing his friend chat to him for a time. The wax rolls, which are quite small and light, will each take five minutes' conversation, and by arranging a cleverly constructed blade fitted to the instrument, an old message can be shaved off a roll while the new one is being spoken to it. The rolls will thus admit of being shaved several times, if it is not desired to keep any message permanently. Some of the songs, etc., heard at St. Mary's last week had been performed two thousand times without any except the initial exertion to the singers. It really looks as though this instrument might entirely revolutionise our present civilisation, for there are a thousand uses to which it can be put in every class of life. It is proposed that a patient should cough into his phonograph at Bournemouth and then send the roll to his London physician to know if he is better. Then, when the medical schools open some October in the future, there will be an announcement that the Sir Andrew Clark to come will address the students of all the schools at the same hour, each in their respective halls. And the patient will never be able to go back on his first statements as he so often does at present. When a man denies that he said he had had rheumatic fever, you have only got to turn on the phonograph and he will give himself the lie. To have one's own voice sounding in judgment against you, to accuse yourself to your face of past misdeeds, is a thought too ghastly for contemplation.

